# Bony Exostosis Development in a Patient Following Free Gingival Graft and Orthodontic Treatment: A Case Report

**DOI:** 10.7759/cureus.50500

**Published:** 2023-12-14

**Authors:** Hussam M Alqahtani

**Affiliations:** 1 Preventive Dental Sciences, College of Dentistry, King Saud Bin Abdulaziz University for Health Sciences, Riyadh, SAU; 2 Research and Development, King Abdullah International Medical Research Center, Riyadh, SAU; 3 Dental Hospital, Ministry of National Guard Health Affairs, Riyadh, SAU

**Keywords:** case report, lesion, orthodontic, free gingival graft, bony exostosis

## Abstract

Free gingival grafts are commonly used to address mucogingival deformities and increase the zone of keratinized tissue. However, the development of bone exostosis after soft tissue graft procedures, such as the free gingival graft, is infrequent. This case report presents the development of a 12x5 mm bony exostosis in the buccal region of the maxillary right canine in a 28-year-old female medical student after a free gingival graft preceding orthodontic treatment. A patient-centric treatment plan, initiated with consultation from an oral pathologist, suggested a biopsy for a definitive diagnosis of a suspected peripheral ossifying fibroma. However, the patient declined due to the absence of symptoms. Therefore, a non-invasive approach involving close monitoring through regular follow-ups has been chosen. In conclusion, this case report highlights the rarity of bony exostosis development after a free gingival graft and orthodontic treatment. The patient's asymptomatic presentation, coupled with the decision to decline the biopsy, underscores the importance of vigilant monitoring. It is significant for dentists to be knowledgeable about this potential complication and its identification to avoid misdiagnosis as oral lesions.

## Introduction

Free gingival graft (FGG) is a predictable procedure to increase the width of keratinized tissue in teeth with subgingival restorative margins prior to orthodontic treatment [1-4]. Stetler and Bissada found that in the presence of a subgingival restoration, the degree of gingival inflammation associated with narrow zones (<2 mm) of keratinized gingiva was significantly greater than in those with ≥2 mm. However, there was no significant difference in unrestored teeth with either wide or narrow zones [2]. In addition, Coatoam et al. found that keratinized gingiva with a width of 2 mm could withstand the stresses of orthodontic mechanics [4]. Although it was previously thought that 2 mm of keratinized tissue was required for gingival health even in the absence of plaque [5], it is now agreed that gingival health can be maintained in areas of narrow keratinized tissue (<2 mm) with appropriate oral hygiene [6-8].

Oliver et al. described the healing of a free gingival graft in three phases: plasmatic circulation in the first three days, revascularization up to 11 days, and maturation up to 42 days [9]. In addition, new bone formation after a free gingival graft has been reported in the literature [10,11]. Pasquinelli used FGG for root coverage and reported that the newly formed bone was 4 mm after 10 months [10]. While there are numerous reports in the literature describing the development of bony exostosis after soft tissue procedures like free gingival grafts [12-14], our report uniquely focuses on bony exostosis following orthodontic treatment and free gingival grafts.

It is crucial to note that bony exostoses of the jaws are prevalent, manifesting as protuberances in various locations, such as torus mandibularis, torus palatinus, or buccal exostoses. Exostoses are typically observed in the 35-65-year age group and are rarely sent for histologic examination as they appear as normal cortical or cancellous bone when examined [15,16]. Their diagnosis involves radiographic and clinical assessments, with a biopsy performed only when suspicion of a syndrome arises or to rule out conditions such as osteosarcomas and chondrosarcomas [17]. The etiology of oral exostoses includes genetic factors [18], environmental influences [19], clenching of jaws, teeth grinding, and bruxism [20]. 

This case report highlights an important finding that has not been previously reported - the development of bony exostosis after orthodontic treatment that was preceded by a free gingival graft. The report emphasizes the significance of dentists being knowledgeable about this potential complication and its identification to avoid misdiagnosis as oral lesions. This new information will increase awareness among dentists and improve patient care. Therefore, this report will present a case of bony exostosis that developed after a free gingival graft was placed prior to orthodontic treatment.

## Case presentation

**Figure 1:**
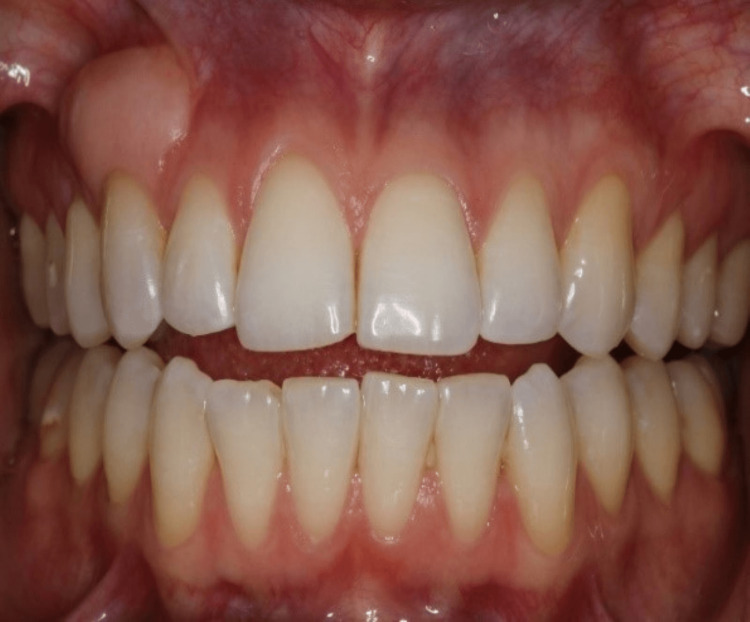
Frontal view of the upper and lower teeth

**Figure 2:**
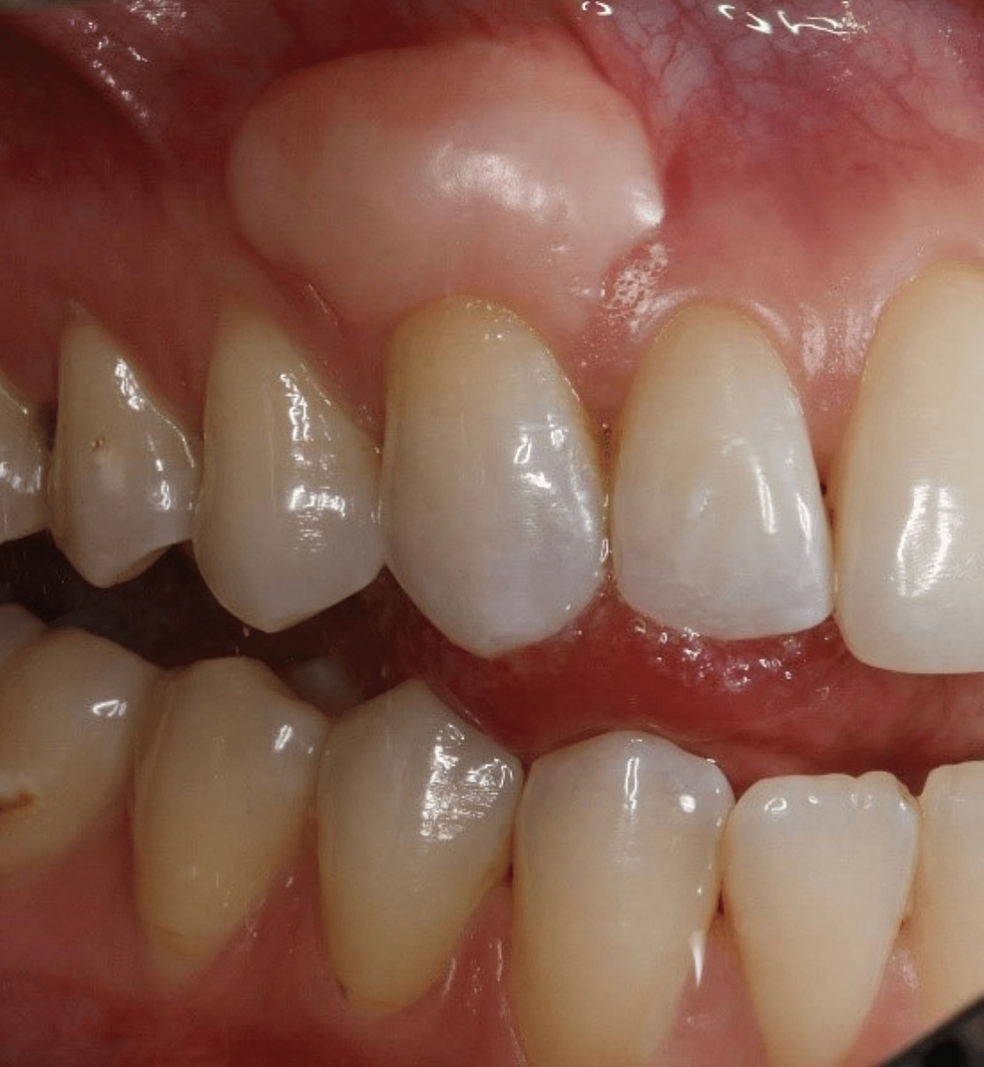
Intraoral view of the developmental bony exostosis

A 28-year-old female medical student with no medical history went to the dental clinic for a checkup and cleaning. On examination by a dental student, a lesion was noted in the buccal area of the maxillary right canine (Figures 1-2). The patient informed us that she visited a periodontist several years ago for free gingival grafts on the maxillary right canine before undergoing orthodontic treatment. Currently, the patient is unaware of the presence of the bony growths and has not reported any complaints.

The dental student consulted an oral pathologist who advised the student to refer the patient to the Department of Oral Maxillofacial Surgery for a biopsy. The oral pathologist's differential diagnosis was peripheral ossifying fibroma. In addition, a resident from the Department of Periodontics was consulted about the lesion. The patient was advised to visit the Graduate Periodontics Clinic for a complete periodontal examination. At the periodontics clinic, the patient stated that she had a free gingival graft placed on her maxillary right canine as a child several years ago as a prerequisite for orthodontic treatment. She also stated that her palate was used to harvest a free gingival graft. Subsequently, orthodontic treatment was performed. She was unaware of the development of the bony exostosis lesion around her upper right canine. 

**Figure 3:**
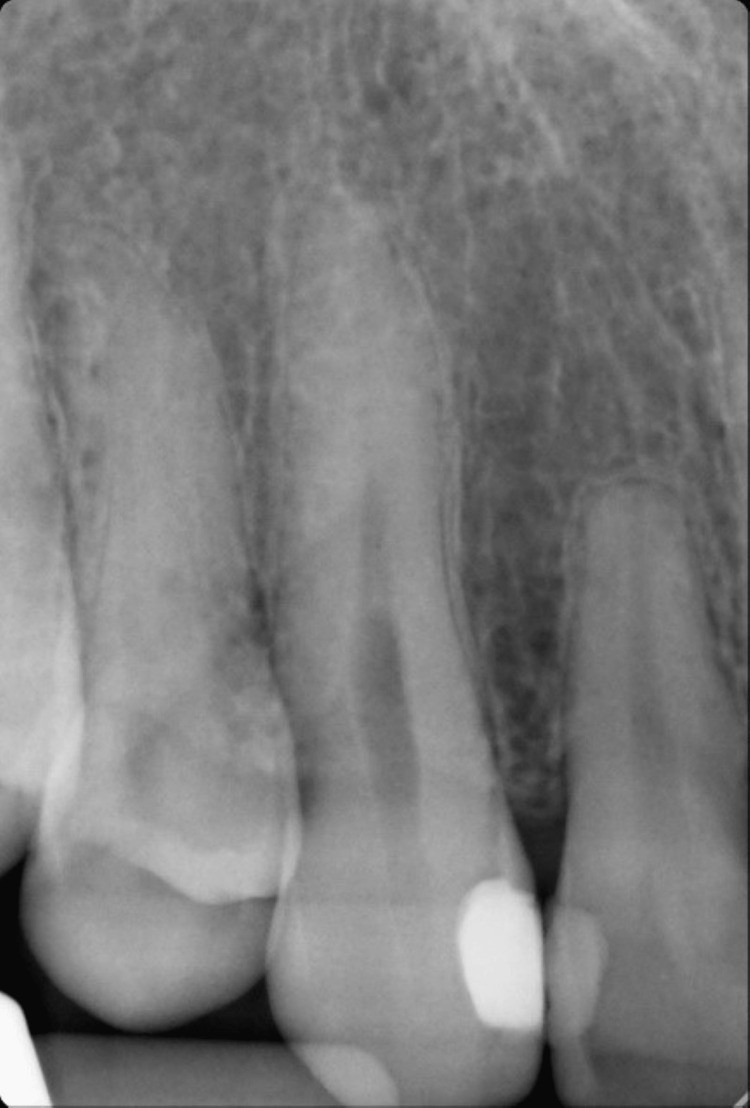
Periapical view of upper right canine with no evidence of pathosis

**Figure 4:**
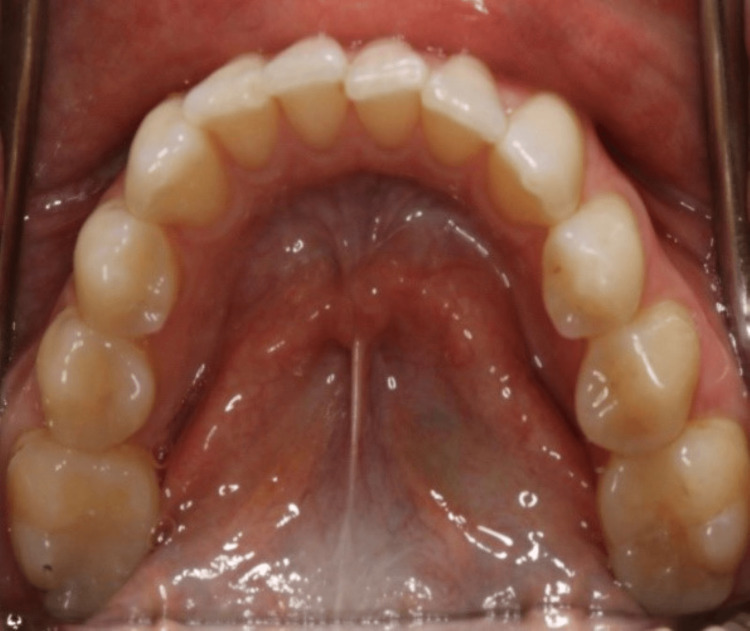
Lingual view of lower teeth with no evidence of bony exostosis

**Figure 5:**
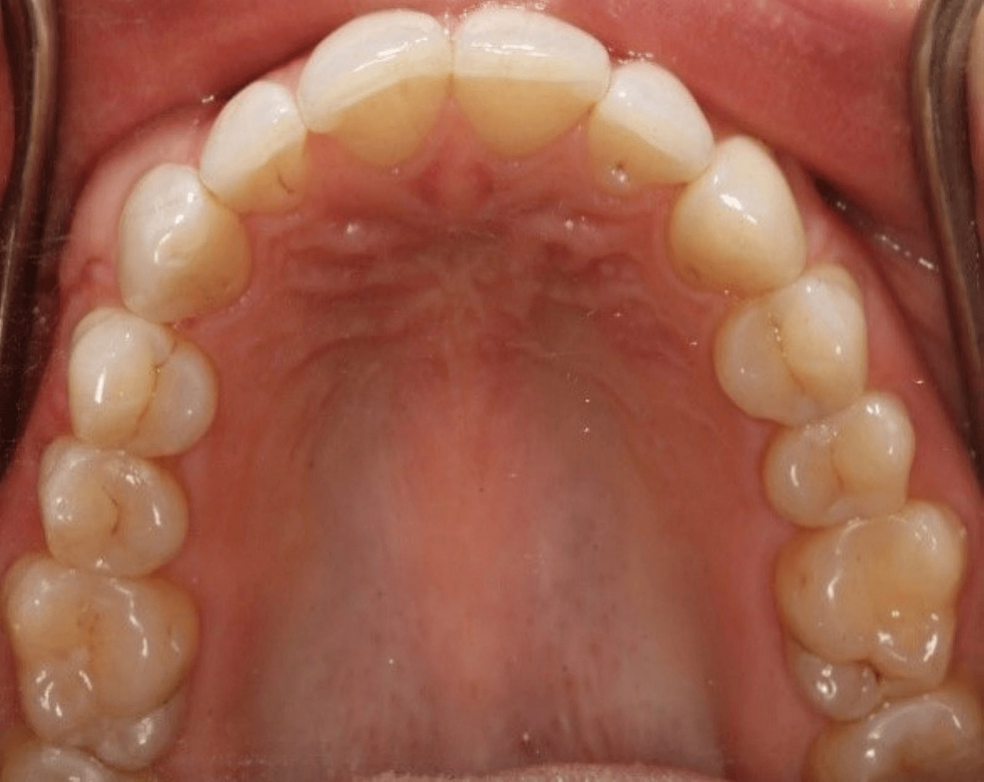
Palatal view of the upper teeth with no evidence of bony exostosis

On clinical examination, the maxillary right canine had a 12x5 mm bony exostosis covered by a wide zone of keratinized attached gingiva (Figure 1). Probing depth was within normal limits, and there was no inflammation of the gingiva. The patient did not notice that the size had increased over the years. She is not concerned about the appearance of the lesion. A radiographic examination revealed that there were no abnormalities (Figure 3). No palatal or mandibular tori were seen in the patient (Figures 4-5). The patient was denied a biopsy for bony exostosis.

The comprehensive treatment plan involved a biopsy for a definitive diagnosis, suspecting a peripheral ossifying fibroma. However, the patient declined the biopsy due to the absence of symptoms. Therefore, a non-invasive approach, including close monitoring through regular follow-ups and educating the patient about the importance of these appointments, has been chosen. The expected outcome involves observing the bony exostosis for any changes, with the possibility of future interventions based on evolving indications. Regular follow-up appointments, integrating clinical and radiographic assessments, are integral to this patient-centered care strategy. As of the latest evaluation, the patient remains asymptomatic, and the 12x5 mm fixed bony exostosis shows no noticeable alterations in size or characteristics. This actual outcome underscores the success of the strategy in maintaining stability and providing valuable insights into the long-term behavior of the bony lesion. Regular follow-up appointments contribute to the ongoing well-being of the patient.

## Discussion

In the presented case, a bony exostosis developed following orthodontic treatment preceded by a free gingival graft. Notably, no observable bony exostosis was present before the orthodontic treatment. Subsequent development of bony exostosis occurred on the upper right side, specifically in the maxillary canine region, after orthodontic treatment. This localization aligns with previous reports indicating a predilection for bony exostosis formation in canines and premolars [14,21].

Periodontal surgery, encompassing graft recipient site preparation, is recognized for inducing trauma to local tissues, activating osteogenic progenitor cells, and resulting in excessive bone growth [12,14,22]. Additionally, potential contributors for the formation of bony exostosis are angiogenic growth of the blood supply after soft tissue grafting, the close association between bone formation and angiogenic growth of blood vessels [23,24], functional stress, genetic factors, chronic irritation, or a combination of periosteal and occlusal influences [22]. Chambrone's insights into the role of elastic fibers and muscle inserts add complexity to the potential etiological factors [14].

The exact processes leading to alveolar bone exostoses subsequent to orthodontic treatment remain uncertain. Agrawal et al. reported bony exostosis occurrence after orthodontic mini-implant placement, attributing it to placement trauma and ongoing orthodontic treatment [22]. Some evidence suggests a correlation with alveolar bone thickening on the labial aspect induced by rapid upper incisor retraction [25]. A previous report highlights a case of a patient developing gingival bony exostoses during orthodontic treatment, particularly after the retraction of upper anterior teeth [26]. Factors influencing changes in alveolar bone thickness during incisor retraction include the rate of tooth movement, buccolingual inclination, the extent of upper incisor intrusion, and the use of orthodontic implants [27,27]. Our study, revealing the development of a bony exostosis after orthodontic treatment preceded by a free gingival graft, contributes to the understanding of these phenomena. Notably, the impact of graft thickness on bone response, particularly when placed on exposed bone, warrants further investigation.

Despite the insights provided by this case report, several limitations must be acknowledged. First, the descriptive nature of the report restricts the identification of potential etiological factors. Second, the patient's refusal of a biopsy due to the absence of symptoms led to a non-invasive monitoring approach. This patient-centric approach provides insights into long-term outcomes, emphasizing the importance of patient-centered care for managing rare occurrences. Third, this case report may be associated with recall bias as the patient provided information about the history of the free gingival graft procedure and orthodontic treatment.

To our knowledge, this case report represents the first documented case of bony exostosis development following a free gingival graft and orthodontic treatment. Given the popularity of orthodontic therapy, it is imperative for orthodontists to be aware of these facts while providing orthodontic care to their patients. In addition, oral pathologists should take note of the study's findings, as they might encounter similar cases and need to distinguish them from other common oral lesions. Moreover, the report underscores the importance of long-term maintenance for periodontic patients who have undergone procedures like free gingival grafts, as they may experience these abnormalities. Further research is needed to clarify the underlying mechanisms and risk factors contributing to the development of bony exostosis in such cases. 

## Conclusions

In conclusion, this case report highlights the development of bony exostosis following a free gingival graft and orthodontic treatment. The patient's decision to decline a biopsy due to the absence of any aesthetic concerns guides us toward a non-invasive approach. This patient-centered strategy emphasizes the importance of personalized care in addressing uncommon situations. It is important to acknowledge the study's limitations for a nuanced interpretation, recognizing that certain aspects of the condition might remain unexplored.
